# Comparative Clinico‐Haematological Alterations of Lumpy Skin Disease Affected Cattle at Different Ages and Periods of Disease in Bangladesh

**DOI:** 10.1002/vms3.70549

**Published:** 2025-08-11

**Authors:** Md. Saiful Islam Siddiqui, Md. Ashiqur Rahman, Saiful Islam, Papri Rani Dey, Md. Khademul Islam, Sultana Begum, Md. Masudur Rahman

**Affiliations:** ^1^ Department of Anatomy & Histology Faculty of Veterinary Animal and Biomedical Sciences Sylhet Agricultural University Sylhet Bangladesh; ^2^ Intern veterinarian Faculty of Veterinary Animal and Biomedical Sciences Sylhet Agricultural University Sylhet Bangladesh; ^3^ MS Fellow Department of Epidemiology & Public Health Sylhet Agricultural University Sylhet Bangladesh; ^4^ Department of Physiology and Pharmacology Jhenaidah Govt. Veterinary College Jhenaidah Bangladesh; ^5^ Department of Pathology Faculty of Veterinary Animal and Biomedical Sciences Sylhet Agricultural University Sylhet Bangladesh

**Keywords:** age category, disease stage, haematology, investigation, lumpy skin disease (LSD)

## Abstract

**Objectives:**

Lumpy skin disease (LSD) is a transboundary viral pox disease that causes huge economic losses. The disease is in an epidemic situation, and the affected cattle show massive lesions and complexity, which is tough to treat; thus, a study was conducted to investigate the clinico‐haematological alterations of LSD‐affected cattle for adopting better pharmacotherapeutic management.

**Material and Methods:**

A total of 36 LSD affected, and 8 healthy/control cattle were examined and used for blood profiling. The control and affected cattle were categorized into four age groups consisting of nine cattle (Group A = under 1 year; Group B = 1 to 2 years; Group C = 2 to 3 years; and Group D = from 4 years up to 10 years) in each group and three disease periods of post infection (prodromal period, period of illness and period of decline) consist of each of 12 LSD affected cattle.

**Results:**

Clinical examination of affected cattle showed 100% cattle (skin nodule of various sizes), 55.55% (lymph node enlargement), 75% (high fever), 13.88% (brisket oedema), 19.44% (limb oedema), 8.33% (ventral abdomen oedema), 5.55% (facial oedema), 19.44% (lameness), 22.22% (respiratory distress), 2.77% (corneal opacity) and 27.78% (salivation and nasal discharge) as clinical signs. Almost all affected cattle showed multiple lesion at the same time, particularly in the last two disease periods. Comparatively, the nodular lesion is much more higher in the limb (33.1%), followed by the head and neck region (23.24%), and then other parts of the body. Haematological analysis reveals a significant decrease in red blood cell (RBC), haemoglobin (Hb), packed cell volume (PCV), mean corpuscular Hb concentration (MCHC), TLC and thrombocytes. During the illness and decline periods, mean corpuscular volume (MCV) increased, which is an indication of macrocytic hypochromic anaemia, lymphocytopenia and thrombocytopenia, and indicative of fluid therapy such as blood transfusion. The age group under 1 year is more vulnerable to LSD fatality, as lower levels of TLC, lymphocyte, and monocyte were found, indicating severe immunosuppression.

**Conclusion:**

It is concluded that disease stage‐wise and age‐wise clinico‐haematological studies have provided baseline data of LSD pathogenesis, which is essential to formulate an effective pharmacotherapeutic strategy.

## Graphical abstract

➢ In comparison, more skin nodules found in head and neck region (23.24%) than other parts of body on the 8th day of infection.

➢ Haematological analysis reveals a significant decrease in RBC, Hb, PCV, MCHC, TLC and thrombocytes.

➢ At the illness and decline periods, MCV increased, which is the indication of macrocytic hypochromic anaemia, lymphocytopenia and thrombocytopenia.

➢ The aged group under one year is more vulnerable to LSD fatality.

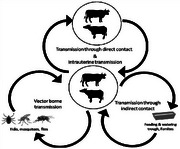

## 1 Introduction

Bangladesh is an agro‐based country and livestock contribution to GDP is about 1.43% and the GDP growth rate of livestock is 3.04% (DLS 2020). Hides are vital raw materials in the leather industry. There are 113 tanneries in the country, producing 180 million square feet of hides and skin, valued at 75 million USD (FAO and UNIDO 2019). Lumpy skin disease (LSD) virus (genus Capripoxvirus and the family Poxviridae) mostly affects cattle health and cattle's hide, causing production loss (meat and milk yield) and lowers of hide's commercial value. The morbidity of LSD ranges from 3% to 85% in different areas and is transmitted mechanically through ectoparasites and arthropods (Ismail and Yousseff 2006). In Bangladesh, as re‐emerging disease, LSD outbreak happened in 2019 as transboundary disease, and Bangladesh shares around 3500 km of bordered area with the neighbouring country, India, and it spread swiftly throughout the country (Hasib et al. 2021).

As severe nodular skin lesions appeared and did not respond to treatment as viral disease; therefore, farmers feel helpless on one hand and, on another sense, face severe economic loss due to huge production loss. Skin and hide exportation is one of the most important means of earning foreign exchange at the country level. To overcome the situation, it is of the utmost need to develop a potent vaccine, which is time‐consuming and expensive. Before that, proper handling and management of the LSD case clinically is the only option to conquer the situation. A Successful clinical management situation depends upon the severity of the disease, that is, disease stages such as viremic stage or third stage or periods of illness condition, as well as gender, immunity and ages of the affected cattle, including individuality. At least no information is available regarding the clinical aspects such as clinical diagnosis, pharmacotherapeutic management, hematobiochemical and tissue alterations of cattle at different ages, and even molecular characterization and phylogenetic analysis of the LSD virus. Therefore, this study was planned to know the complete blood profile of LSD‐affected cattle, including differential leukocyte count at different age categories, as well as to investigate the most vulnerable age group of cattle, which will facilitate an easy approach for prompt clinical diagnosisand the most effective pharmacotherapeutic management of LSD‐affected cattle.

## 2 Materials and Methods

### 2.1 Study Area and Duration

The investigation was conducted from May 2023 to February 2024. During that period, samples were collected intermittently from various veterinary hospitals in Sylhet Sadar and from the Veterinary Teaching Hospital at Bangladesh Agricultural University in Mymensingh. The study was conducted in an areas where outbreak of LSD is reported and animals are continuously affected by LSD. For the control group, samples were collected from healthy cattle of Sylhet and a crossbreed cow from the Suparibagan area of Boyra, Sadar Upazilla, Mymensingh—where LSD outbreak is not reported, and animals that are devoid of natural LSD infection.

### 2.2 Study Animals and Population

A total of 36 LSD‐suspected cattle of different ages and disease stages and 8 healthy cattle of different ages as controls were included in this study. LSD affected animals (those served our study purpose) were identified clinically, histories were recorded, tagged and kept under close and continuous observation, related data were taken and recorded and preserved, and blood samples were obtained following study design.

### 2.3 Experimental Design

#### 2.3.1 Age‐Wise Categorization of the Animals

A total of 36 LSD‐affected cattle age ranges between 3 months and 10 years were randomly employed in the study, consisting of 14 males and 22 females. These Cattle are divided into 4 age groups (Group A = under 1 year; Group B = 1 to 2 years; Group C = 2 to 3 years; and Group D = 4 years up to 10 years). Blood was collected and used for haematological tests, and then data were analysed for investigation of the age‐wise haematological alterations or effect of LSD‐affected cattle in contrast to the controlled group. Each age group consists of nine LSD‐affected cattle. Both infected and control group animals matched in this study were almost under same age, breed, health status, or environmental conditions and management.

#### 2.3.2 Categorization of LSD‐Affected Cattle Based on Disease Periods

Though this categorization has some limitations but based on clinical suspicion, lesion characterization, history of the disease, and time duration LSD‐affected study cattle (*N* = 36) were categorized into three disease period groups following the modified protocol of Coffey and Goldfarb (1986) and Gonnella and Louis (1987 and 1998). Each period consists of 12 LSD‐affected cattle. Ages of the cattle, body score, husbandry and managemental issues were considered seriously during this categorization.

- **Prodromal period—**Body temperature of LSD‐affected cattle nearby normal with the first appearance of skin lesion. Symptoms are too general to indicate a disease. Cross‐examination revealed soreness, pain, inflammation in the skin and a continuous increase in body temperatures. Only the cattle having signs and symptoms clinically suggestive of LSD and later those who were clearly shown all signs and symptoms of LSD were considered for this study.
- **Period of illness—**Symptoms of the disease are most obvious, specific and severe. LSD affected cattle with high temperature, indicating viremia, severe and more skin lesions involved multiple sites with other clinical conditions, confirmed by the cross‐examination.
- **Period of decline**—Cattle with a history of clinical signs begin to decline with subsided temperature.

### 2.4 Clinical Study of the Studied Cattle

A close clinico‐physical examination of the affected cattle was conducted, including the history of nodules over the body (first appearance of skin lesion, areas involved, nodular lesion development and distribution, nodular size, number of nodules, the relation of nodules development with the raising of body temperature and time of subsiding skin lesion), rectal temperature and palpation of superficial lymph nodes. Swelling of limbs, brisket oedema, lameness, nasal discharge, lacrimation and lumps on various sites of the body, reduced feed and water intake, salivation, brisket oedema and lameness were observed, studied, measured and recorded. Lesion types were categorized on the basis of clinical observation across three disease progression stages: prodromal (early signs prior to full illness), periods of illness (peak clinical signs) and period of decline (late‐stage or recovery). Frequency (%) of various clinical signs observed in cattle during different disease stages (prodromal, illness and decline; *N* = 12). Lesions and symptoms were recorded on the basis of daily clinical monitoring. Animals were classified into disease stages according to the onset, peak and resolution of clinical symptoms.

### 2.5 Sample (Blood) Collection, Preservation (Blood Bank) and Transportation to the Laboratory

After tentative diagnosis of the disease, with proper restraining of the cattle, blood is collected from 36 cattle by jugular vein puncture using disposable syringe and preserved in 2 mL K2 EDTA tube for haematological test. Body temperature was taken before collecting blood with both digital and analogue thermometers, and the samples were categorized as per study cattle ages and disease periods wise categorization.

### 2.6 Laboratory Test for Complete Blood Count (CBC)/Complete Haematological Profile

For CBC including differential leukocyte count the automated haematology analyser ‘Sysmex XT‐2000iV’ was used following the modified protocol of Bauer et al. (2011).

### 2.7 Statistical Analysis

All data were noted initially in Microsoft Excel for Windows 11. *T*‐test was done for comparison among the age group and disease stage category and to find the significance of those differences (ANOVA) was performed by using SPSS 25.0 for Windows 11.

## 3 Results

### 3.1 Clinical Findings

#### 3.1.1 Frequency of Different Clinical Signs Among LSD‐Affected Cattle

**FIGURE 1:**
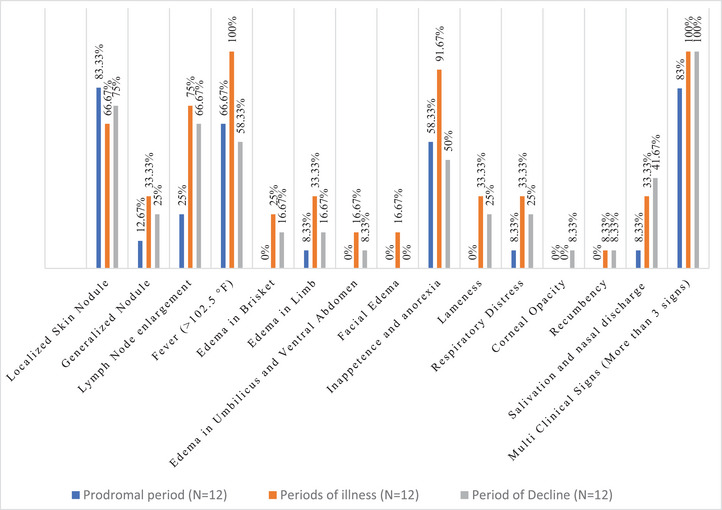
Frequency of different clinical signs among LSD‐affected cattle at different periods of disease (Total LSD‐affected cattle, *N* = 36; Number of LSD‐affected cattle at **Prodromal period**, *n* = 12, at **Period of illness,** *n* = 12 and at **Period of decline**, *n* = 12).

Out of 36 LSD‐affected cattle, 12 were in the p**rodromal period**, 12 were in the p**eriod of illness** (Days 4–12), and another 12 were in the p**eriod of decline**. Two cattle had died of the 36 affected cattle. The affected LSD cattle displayed clinical signs such as high fever, skin nodules, lymph node enlargement, anorexia, oedema, respiratory distress, lameness, corneal opacity and recumbence. Data were categorized on the basis of observational clinical scoring. The frequency of different clinical signs among LSD‐affected cattle at different periods of disease is shown in Figure 1.

#### 3.1.2 Distribution of the Average Number of Localized Skin Nodules on Different Regions and Parts of the Body of LSD‐Affected Cattle

**FIGURE 2:**
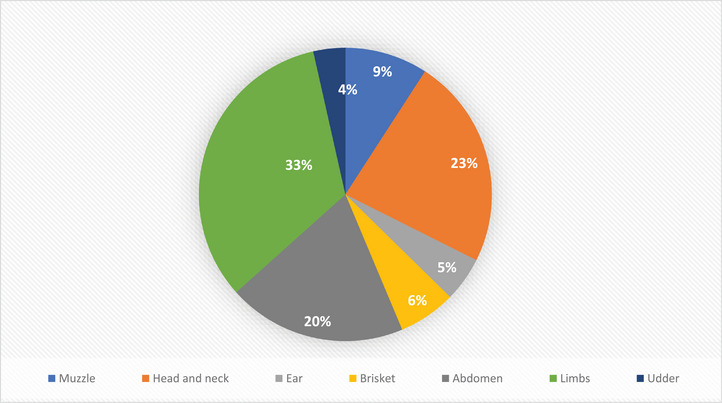
Distribution of the average number of localized skin nodules on different regions and parts of the body of LSD‐affected cattle at period of illness of disease.

Localized skin nodules were found in different areas or parts of the body of LSD‐affected cattle but were predominant in the limb (33.1%), followed by the head and neck (23.24%) and abdomen (19.72%). The distribution of localized skin nodules in other parts of the body is shown in Figure 2.

### 3.2 Haematological Findings of LSD Affected Cattle Among Three Periods of Disease

Haematological investigation of LSD‐infected cattle at three distinct periods of disease was performed. LSD‐infected cattle showed a significant reduction of red blood cells (RBCs), haemoglobin (Hb), packed cell volume (PCV) and mean corpuscular Hb concentration (MCHC) compared to the normal or reference values found in cattle of the control group. Values of other blood parameters such as white blood cells (WBCs), lymphocytes and basophils were also found in reduction than reference range. Erythrocyte sedimentation rate (ESR) is much higher in LSD‐affected cattle compared to the reference value. Insignificant reduction of MCH, eosinophils and platelets were found. Monocytes and mean corpuscular volume (MCV) did not show significant alterations, although they experienced an increase in number. Table 1 displays the altered value of different haematological parameters of LSD‐infected cattle at different periods of disease.

**TABLE 1:** Alteration in blood parameters at different periods of natural lumpy skin disease (LSD)‐infected cattle (mean values ± SE).

			LSD affected cattle			
Sl. No.	Parameter	Healthy control group (*N* = 8)	Prodromal period (*N* = 12)	Period of illness (*N* = 12)	Period of decline (*N* = 12)	*p* value
1	RBC count (g/dL)	9.23 ± 0.71	7.36 ± 1.35	6.90 ± 1.37	6.54 ± 1.31	0.0001^***^
2	HB (g%)	13.45 ± 1.11	10.3 ± 1.99	9.32 ± 1.39	9.42 ± 1.36	0.0001^***^
3	PCV (%)	40.63 ± 2.72	32.1 ± 4.71	30.99 ± 3.67	31.58 ± 3.61	0.0001^***^
4	MCV (fL)	44.11 ± 0.81	44.24 ± 6.39	47.19 ± 8.37	49.60 ± 9.90	0.284 NS
5	MCH (pg)	14.70 ± 0.45	14.1 ± 1.84	13.77 ± 2.01	14.68 ± 2.22	0.577 NS
6	MCHC (g/dL)	33.26 ± 0.62	32.11 ± 2.64	29.83 ± 3.77	29.84 ± 2.73	0.019^*^
7	TLC count (10^3/µL)	12.03 ± 0.56	8.37 ± 1.57	8.09 ± 2.58	8.86 ± 2.43	0.001^**^
8	Neutrophil (10^3/µL)	4.18 ± 0.42	2.82 ± 0.75	2.95 ± 1.48	3.17 ± 1.33	0.062 NS
9	Eosinophil (10^3/µL)	0.04 ± 0.05	0.04 ± 0.06	0.05 ± 0.05	0.06 ± 0.07	0.898 NS
10	Basophil (10^3/µL)	0.01 ± 0.01	0.02 ± 0.02	0.03 ± 0.03	0.02 ± 0.01	0.053 NS
11	Lymphocyte (10^3/µL)	7.40 ± 0.41	4.99 ± 0.91	4.60 ± 1.11	5.33 ± 1.31	0.0001^***^
12	Monocyte (10^3/µL)	0.43 ± 0.14	0.52 ± 0.22	0.48 ± 0.13	0.55 ± 0.19	0.464 NS
13	Platelets (10^6/µL)	412.50 ± 88.44	346.83 ± 130.66	330.42 ± 139.30	284.08 ± 102.42	0.147 NS
14	ESR mm/h	7.50 ± 2.67	12.92 ± 4.51	13.75 ± 5.28	15.35 ± 3.98	0.003^**^

Abbreviations: ESR, erythrocyte sedimentation rate; Hb, haemoglobin; MCH, mean corpuscular haemoglobin; MCHC, mean corpuscular haemoglobin concentration; MCV, mean corpuscular volume; PCV, packed cell volume; RBCs, red blood cells; SE, standard error; TLC, total leucocyte count.

* *p* < 0.05

** *p* < 0.01

*** *p* < 0.001

### 3.3 The Relationship Between the Findings From Clinical Cases With That of Haematological Results

This study revealed that major clinical signs (multiple signs—at least, more than three) were found predominant at period of illness (Figure 1), when haematological values was found significantly altered. Highest percentage (100%) of affected cattle's showed high temperatures clinically at the period of illness, with lowest total leukocyte count (8.09 ± 2.58) indicates severe leucopenia (Figure 1 and Table 1). Highest percentage (75%) affected cattle showed maximum lymph node enlargement with lowest lymphocyte count, lymphocytopenia (Figure 1 and Table 1).

### 3.4 Haematological Findings of LSD Affected Cattle in Different Age Groups

LSD‐affected cattle were divided into four age groups, and haematological investigations at different age groups were conducted to find out the most vulnerable age group of cattle to LSD infection. The analysis indicates substantial differences in RBC, HB, PCV, TLC, lymphocytes, monocytes, platelets and ESR compared to the control group. Cattle of age Group A (under 1 year) have lower levels of PCV, TLC, lymphocytes, monocytes and ESR compared to the other three age groups. Cattle of Group B (1–2 years) display elevated MCV and mean corpuscular Hb (MCH), along with decreased neutrophil count compared to the other three age groups. Cattle of Group C (2–3 years) exhibited a higher ESR compared to the other groups. Cattle of age Group D (4 years and above) showed decreased levels of RBCs, Hb, MCHC and platelets compared to others (Table 2).

**TABLE 2:** Alteration in blood parameters at different age groups of naturally lumpy skin disease (LSD)‐infected cattle (mean values ± SE).

			LSD affected cattle				
Sl. No.	Parameter	Control group (*N* = 8)	Age Group A (Under 1 year) *N* = 9	Age Group B (1–2 years) *N* = 9	Age Group C (2–3 years) *N* = 9	Age Group D (4–10 years) *N* = 9	*p* value
1	RBC Count (g/dL)	9.23 ± 0.71	7.11 ± 0.37	6.87 ± 0.58	7.20 ± 0.39	6.54 ± 0.48	0.001^***^
2	HB (g%)	13.45 ± 1.11	9.54 ± 0.62	10.18 ± 0.66	9.68 ± 0.55	9.31 ± 0.30	0.0001^***^
3	PCV (%)	40.63 ± 2.72	30.14 ± 1.81	33.78 ± 1.23	30.82 ± 1.09	31.4 ± 0.77	0.0001^***^
4	MCV (fL)	44.11 ± 0.81	43.49 ± 1.18	51.24 ± 3.53	43.32 ± 1.60	49.97 ± 3.40	0.054 NS
5	MCH (pg)	14.70 ± 0.45	13.43 ± 0.46	15.15 ± 0.80	13.46 ± 0.34	14.67 ± 0.84	0.151 NS
6	MCHC (g/dL)	33.26 ± 0.62	31.82 ± 1.22	29.96 ± 1.09	31.25 ± 0.86	29.34 ± 1.02	0.061 NS
7	TLC count (10^3/µL)	12.03 ± 0.56	8.05 ± 1.03	8.37 ± 0.93	8.70 ± 0.37	8.67 ± 0.51	0.002^***^
8	Neutrophil (10^3/µL)	4.18 ± 0.42	3.08 ± 0.59	2.82 ± 0.47	3.02 ± 0.16	3.01 ± 0.31	0.138 NS
9	Eosinophil (10^3/µL)	0.04 ± 0.05	0.03 ± 0.01	0.06 ± 0.02	0.05 ± 0.02	0.07 ± 0.024	0.669 NS
10	Basophil (10^3/µL)	0.01 ± 0.01	0.013 ± 0.001	0.02 ± 0.008	0.02 ± 0.007	0.03 ± 0.01	0.153 NS
11	Lymphocyte (10^3/µL)	7.40 ± 0.41	4.54 ± 0.46	4.91 ± 0.42	5.10 ± 0.24	5.33 ± 0.38	0.0001^***^
12	Monocyte (10^3/µL)	0.43 ± 0.14	0.38 ± 0.04	0.56 ± 0.07	0.52 ± 0.05	0.62 ± 0.05	0.027^*^
13	Platelets (10^6/µL)	412.50 ± 88.44	398.11 ± 57.67	302.89 ± 39.92	314.67 ± 33.03	266.11 ± 18.78	0.048^*^
14	ESR mm/h	7.50 ± 2.67	11.67 ± 1.86	14.91 ± 0.84	15.0 ± 1.67	14.44 ± 1.55	0.010^**^

Abbreviations: ESR, erythrocyte sedimentation rate; Hb, haemoglobin; MCH, mean corpuscular haemoglobin; MCHC, mean corpuscular haemoglobin concentration; MCV, mean corpuscular volume; PCV, packed cell volume; RBCs, red blood cells; SE, standard error; TLC, total leucocyte count.

* *p* < 0.05

** *p* < 0.01

*** *p* < 0.001

*P* value displays significant differences (*p* < 0.05) in the PCV (%), MCH (pg), and the number of monocytes between Groups A and B. Groups B and C experience a significant difference in MCV and MCH count, and the number of monocyte and platelet count reveals a significant difference between Groups A and D (Table 3).

**TABLE 3:** Statistical difference of haematological parameters among four aged grouped lumpy skin disease (LSD)‐affected cattle (*p* value).

Sl. no.	Parameter	LSD‐affected cattle					
		Groups A and C	Groups A and D	Groups B and C	Groups B and D	Groups C and D	Groups A and C
1	RBC count (g/dL)	0.692	0.878	0.358	0.583	0.598	0.285
2	HB (g%)	0.391	0.856	0.751	0.497	0.242	0.618
3	PCV (%)	**0.039** ^*^	0.692	0.449	0.090	0.177	0.716
4	MCV (fL)	0.183	0.961	0.067	**0.027** ^*^	0.714	0.060
5	MCH (pg)	**0.048** ^*^	0.970	0.146	**0.052** ^*^	0.577	0.156
6	MCHC (g/dL)	0.184	0.681	0.079	0.354	0.653	0.172
7	TLC count (10^3/µL)	0.750	0.521	0.531	0.745	0.756	0.988
8	Neutrophil (10^3/µL)	0.646	0.916	0.899	0.723	0.738	0.984
9	Eosinophil (10^3/µL)	0.308	0.513	0.173	0.712	0.724	0.471
10	Basophil (10^3/µL)	0.451	0.589	0.238	0.829	0.666	0.517
11	Lymphocyte (10^3/µL)	0.468	0.268	0.121	0.699	0.400	0.647
12	Monocyte (10^3/µL)	**0.030** ^*^	0.098	**0.004** ^**^	0.579	0.430	0.183
13	Platelets (10^6/µL)	0.079	0.122	0.**017** ^*^	0.824	0.489	0.363
14	ESR mm/h	0.121	0.111	0.183	0.966	0.821	0.787

Abbreviations: ESR, erythrocyte sedimentation rate; Hb, haemoglobin; MCH, mean corpuscular haemoglobin; MCHC, mean corpuscular haemoglobin concentration; MCV, mean corpuscular volume; PCV, packed cell volume; RBCs, red blood cells; TLC, total leucocyte count. Bold values mean significant at 5% and 1% level of probability w

* *p* < 0.05

** *p* < 0.01

## 4 Discussion

The investigation initially showed that localized nodules were present in 83.33% of cases, whereas generalized nodules were discovered in only 12.67% of cases. During the period of illness (Days 4–12), 33.33% of the infected cattle displayed generalized nodules across their bodies, whereas 66.67% showed localized nodules. During the recovery period, 75% of cattle exhibited localized nodules, whereas 25% showed generalized nodules and these nodules started to heal after rupture. The present clinical findings recorded in the study agreed with earlier reports of Prozesky and Barnard (1982) and Tuppurainen et al. (2017). In this study, 75% of cases had expansion of the superficial lymphoid glands (mandibular, femoral and parotid lymph nodes) during the viremia stage. Similar findings were noticed by Prozesky and Barnard (1982). The LSD virus gets into the regional lymph node and leads to lymphadenitis (Mat et al. 2021). The primary and most noticeable clinical symptom is fever. Cattle exposed to LSD exhibited high fever of 104–106°F due to the quick release and expulsion of pyrogens (Ismail and Yousseff 2006). Within the first 3 days, 66.67% of cattle exhibited high fever (>102.5°F), with the prevalence increasing each day thereafter. Between Days 4 and 12, 91.67% of cattle experienced high fever (>102.5°F), which decreased approximately after 12 days. Additionally, 58.33% of cattle sustained a fever (>102.5°F) at the late/decline stage (up to 20 days of recovery), which is likely linked to subsequent bacterial infection and is in accordance with the study findings of Agag et al. (1992) and Coetzer et al. (1994). Approximately 58.33% of the affected cattle showed anorexia in the initial 3 days, which escalated with high fever and widespread nodules, peaking at 91% between Days 4 and 12. Following the period of illness, there is a progressive reduction of nodule sizes and spread. Regarding the percentage of nodules revealed, maximum nodules were found on the 8th day of affection, measured by the cattle owner's commentary in the limbs region and it was one‐third of all the nodules and consequently, the abdomen (20%), head and neck region (23%), muzzle (9%), brisket (6%), ear (5%) and udder (4%) nodule were observed.

Regarding the erythrogram results revealed, a non‐significant change in RBC, MCV, MCH and MCHC within 1st–3rd day post affection which agrees with the findings of Neamat‐Allah (2015) but a significant change in Hb was observed, which does not follow Neamat‐Allah (2015). At the 4–12th day and late‐stage of LSD, post‐infection revealed a significant decrease in RBCs, Hb and PCV, which is in accordance with Neamat‐Allah (2015). This result was interpreted as a macrocytic hypochromic anaemia, which is in agreement with those who reported that haemolytic anaemia could have occurred with viral infection (Brooks et al. 2022). Decreasing the number of RBCs is not unnatural due to the impact of the dehydration condition of the cattle and the pathogenic mechanism of erythropoiesis during viral infection. MCHC is decreased with a significant increase in MCV when compared to the healthy control group, which is not the same as described as Neamat‐Allah (2015). The TLC and the percentage of neutrophils and monocytes were lower in affected cattle as compared to the healthy ones, which is similar to the result of Abutarbush (2015), but differs from the result of Neamat‐Allah (2015). Similar to our findings, El‐Mandrawy et al. (2018) reported significant differences in TEC and Hb levels of LSD‐infected cattle when compared to healthy ones. The lymphocyte count was higher in healthy cattle (70.40%) than in LSD‐affected cattle (57.40%). Thrombocytopenia was identified in all affected cattle, characterized by a drop in platelet count caused by bone marrow abnormalities. Thrombocytopenia may occur due to increased platelet usage caused by disseminated intravascular coagulation (DIC) and systemic vasculitis resulting from LSDV's preference for endothelial cells (El‐Neweshy et al. 2013; Sanz‐Bernardo et al. 2020). All the infected cattle exhibited thrombocytopenia, which may result from diminished platelet production induced by bone marrow abnormalities or platelet sequestration, which can occur in conditions including splenomegaly and non‐infectious inflammatory disorders (Ul‐Rahman et al. 2023). A shortened platelet lifetime is the primary cause of thrombocytopenia in cattle. This is typically the result of increased platelet usage induced by DIC, severe endotoxemia and systemic vasculitis. In this study, thrombocytopenia is likely caused by extensive vasculitis, which is a hallmark histopathological finding of LSD due to its tropism for endothelial cells (Abutarbush 2015). Erythrogram variation also found among different age groups, RBC count is lower at aged Group D (6.54 ± 0.48 g/dL) and after that, consequently, Group B (6.87 ± 0.58 g/dL), Group A (7.11 ± 0.37 g/dL) and Group C (7.20 ± 0.39). All the aged grouped cattle showed anaemic condition at the time of LSD virus infection. LSD‐affected aged Group A (under 1 year) cattle shows lower levels of PCV (30.14% ± 1.81%), TLC (8.05 ± 1.03 × 10^3^ µL), lymphocytes (4.54 ± 0.46 × 10^3^ µL) and monocytes (0.38 ± 0.04 × 10^3^ µL) compared to the other three aged group LSD affected cattle. Aged Groups A and D are vulnerable because of leucopenia, lymphocytopenia, macrocytic hypochromic anaemia, which is similar to the result of Haque and Gofur (2020). Decreasing number of leukocytes causes the weaker of the immune system to the affected calf. It also causes increased susceptibility to the secondary bacterial infection. Other aged group has a significant impact on LSDV like leucopenia, lymphopenia and a significant production loss, which follows the result of Neamat‐Allah (2015) and Hasib et al. (2021). According to the result, there are no significant difference between different aged grouped affected cattle among haematological values: RBC, Hb, MCHC, TLC, Neutrophil, eosinophil, basophil and leukocyte count, which is similar to the result of Bedenicki et al. (2014). There were only found a significant difference at the amount of PCV%, MCH and monocytes in between aged Group A and aged Group B; monocyte and platelets count in between aged Group A and aged Group D; and at last MCV count in between aged Group ‘B’ and aged Group ‘C’ which is difference to the result of Bedenicki et al. (2014). Aged Group A (under 1 year) is most vulnerable, and that's why it shows significant differences from other aged grouped LSD‐affected cattle. The reasons behind this are not known but may be immunosuppression due to leucopenia, which might be confirmed by advanced studies regarding assessment of immunity, though this has not been done in this study, though there are also some limitations of disease staging or periods categorization in natural infection, as disease starting could not calculate easily. Despite having few limitations, different periods of disease is considered LSD takes a long time to recover. For formulation of an effective pharmacotherapeutic strategy, disease periods can be considered strategic tools to find out the blood parameters alterations, which might have positive impact on affected animal health management. Therefore, here the modified protocol of disease staging (Coffey and Goldfarb 1986; Gonnella and Louis 1987) is followed.

## 5 Conclusion

LSDV‐infected cattle at the periods of illness exhibit leucopenia, necessitating immunostimulant treatment. The late‐stage (periods of decline) presentation included haemolytic anaemia and leukocytosis, necessitating blood transfusion and fluid therapy. The main clinical symptoms in cases of LSD in cattle of all ages include macrocytic hypochromic anaemia, leucopenia and thrombocytopenia indicative to fluid therapy such as blood transfusion. These symptoms are probably caused by the intense inflammatory processes and illness consequences, leading to issues like anorexia and decreased muscle mass. Under the age of 1 year, cattle is most vulnerable because of leukocytopenia and as a consequence of secondary bacterial infection. It is concluded that disease period‐wise and age‐wise clinico‐haematological alterations have provided baseline data of LSD pathogenesis, which is essential to formulate an effective pharmacotherapeutic management strategy.

## Author Contributions

**Md. Saiful Islam Siddiqui**: conceptualization, investigation, funding acquisition, writing – original draft, methodology, validation, visualization, project administration, formal analysis, software, supervision, resources. **Md. Ashikur Rahman**: conceptualization, methodology, data curation, writing – original draft, investigation. **Saiful Islam**: investigation, validation, data curation, writing – original draft. **Papri Rani Dey**: investigation, validation, formal analysis, data curation. **Md. Khademul Islam**: conceptualization, investigation, methodology, data curation, formal analysis. **Sultana Begum**: investigation, validation, formal analysis, supervision. **Md. Masudur Rahman**: investigation, validation, formal analysis, supervision, project administration, funding acquisition, resources.

## Ethics Statement

All methods were carried out in accordance with relevant guidelines and regulations of the ‘Animal Experimentation and Ethics Committee’, Sylhet Agricultural University, Bangladesh The approved Animal Use Protocol No. was [#AUP2023032] for conducting the experiment. Confirm that all methods are reported in accordance with ARRIVE guidelines (https://arriveguidelines.org). The Source of the experimental animals were local market.

## Conflicts of Interest

The authors declare no conflicts of interest.

## Peer Review

The peer review history for this article is available at https://publons.com/publon/10.1002/vms3.70549.

## Data Availability

All data generated or analysed during this study are included in this published article (and its Supporting Information files).
